# Association of female genital schistosomiasis and human papillomavirus and cervical pre-cancer: a systematic review

**DOI:** 10.1186/s12905-024-03514-0

**Published:** 2025-01-03

**Authors:** Amy Sturt, Tanvier Omar, Isaiah Hansingo, Paul Kamfwa, Amaya Bustinduy, Helen Kelly

**Affiliations:** 1 Infectious Diseases Section, Veterans Affairs Health Care System, Palo Alto, CA USA; 2 Department of Infectious Diseases and Geographic Medicine, Stanford University, 300 Pasteur Drive, Lane Building 134, Stanford, CA 94025 USA; 3 Faculty of Health Sciences, University of Witwatersrand, Johannesburg, South Africa; 4 Department of Obstetrics and Gynaecology, Livingstone Central Hospital, Livingstone, Zambia; 5 Department of Gynecology Oncology, Cancer Diseases Hospital, Lusaka, 10101 Zambia; 6 Department of Clinical Research, London School of Hygiene and Tropical Medicine, London, UK

**Keywords:** Female genital schistosomiaisis (FGS), *Schistosoma haematobium*, Human papillomavirus (HPV), Pre-cancer, Cervical cancer

## Abstract

**Background:**

*S. haematobium* is a recognized carcinogen and is associated with squamous cell carcinoma of the bladder. Its association with high-risk(HR) human papillomavirus (HPV) persistence, cervical pre-cancer and cervical cancer incidence has not been fully explored.

**Methods:**

We searched OvidSP MEDLINE, OvidSP Embase, Global Index Medicus, PubMed and the Wiley Cochrane library without date or language restrictions up to April 20, 2024 for abstracts evaluating the association of female genital schistosomiasis (FGS) with the prevalence, incidence or persistence of cervical HR-HPV, and incidence of histology-verified cervical pre-cancer or cancer. Cervical pre-cancer defined using cervical cytology or visual inspection with acetic acid (VIA) was also considered, but as lower quality evidence. We assessed the risk of bias of included studies using a modified Newcastle Ottawa scale. This study is registered on PROSPERO: CRD42023389301.

**Results:**

We identified 1,170 publications and six studies were eligible for inclusion. Five studies were cross sectional and 1 was prospective. The studies describe 1081 women living in sub-Saharan Africa. One study from Zimbabwe reported an increased risk of HR-HPV prevalence at baseline in women with composite-FGS compared to women without FGS (aOR 1.9, 95% CI 1.1 – 3.6, *p* = 0.03), however no association was seen after 5 years of follow-up. Another study from KwaZulu-Natal reported an increased odds of any HPV prevalence among women with visual-FGS compared to women without FGS (aOR 1.71 [1.14 – 2.56], *p* = 0.01). However, a study in Madagascar did not show increased odds of any HPV among women with visual-FGS compared to women without FGS (OR 1.0 [0.82 – 1.2). Of 4 studies evaluating the association of FGS and cervical pre-cancer, one reported an increased risk of VIA abnormalities in women with molecular-FGS compared to those without (aOR 6.08, 95% CI 1.58 – 23.37). Three studies did not report an association between FGS and cervical pre-cancer (cytology defined (*n* = 2) and histology defined (*n* = 1)).

**Conclusion:**

There are limited and low quality data on the risk of HR-HPV infection and cervical pre-cancer and cancer among women with FGS. Given limited data, it was not possible to confirm or exclude an association between FGS and HPV, cervical pre-cancer, and cervical cancer and additional research is needed.

**Supplementary Information:**

The online version contains supplementary material available at 10.1186/s12905-024-03514-0.

## Introduction

Cervical cancer remains a major public health problem despite being preventable and curable, if diagnosed early and with access to effective treatment. In 2018, the World Health Organization (WHO) called for the elimination of cervical cancer to address this public health issue and member states adopted WHO’s Global strategy to accelerate the elimination of cervical cancer in 2020. The targets to achieve elimination are based on vaccination of 90% of girls by age 15; screening of 70% of women twice by age 35 and 45 with a high-performance test; and treatment for 90% of women identified with cervical pre-cancer or invasive cancer [1]. Persistent infection with high-risk (HR) human papillomavirus (HPV) (types 16, 18, 31, 33, 35, 39, 45, 51, 52, 56, 58, 59) is necessary for the development of cervical cancer [2, 3]. While persistent infection is necessary for the development of pre-cancerous lesions, other factors also increase a woman’s risk of developing pre-cancerous lesions [4].

Cervical cancer is one of the most preventable cancers due to the availability of effective tools for prevention. Screening and treatment programmes using visual inspection methods, cervical cytology and HPV-DNA for screening, and ablative and excisional methods for treating cervical precancer have shown to be effective in preventing cervical cancer [5]. HPV vaccination of girls and young women prior to their sexual debut is highly effective at reducing risk of cervical cancer [6]. Current available HPV vaccines can target up to 7 high-risk, or oncogenic HPV types (HPV16/18/31/33/45/52/58) that protect against 90% of cervical cancers [7]. The high incidence of cervical cancer in low and middle income countries is associated with limited access to HPV vaccination [8], nationally organised, population-based cervical cancer screening and treatment programmes [9], and the impact of HIV [10]. However, other exposures may be co-factors in the high incidence of cervical cancer in low and middle-income countries. Over 160 million people are estimated to have schistosomiasis in sub-Saharan Africa [11], a region with the highest global cervical cancer incidence [12]. The parasite *Schistosoma (S.) haematobium* is recognized by the International Agency for Research on Cancer (IARC) as a carcinogen and is associated with squamous cell carcinoma of the bladder [13, 14] but the parasite’s role as a co-factor in cervical cancer has not been fully explored.

*S. haematobium* is a blood fluke that is often endemic in areas with limited access to clean water, sanitation and hygiene resources [15]. *S. haematobium* infection occurs when exposed skin contacts parasite cercariae in fresh water [16]. Adults and children of both sexes may be exposed to *S. haematobium* differently based on how local household division of labor intersects with fresh water contact [17]. Once inside the human body, the immature *S. haematobium* parasite circulates until transforming into adults that migrate through the venous system to commence egg-laying [18]. The majority of *S. haematobium* eggs will be excreted through the urinary bladder, but given the proximity of the vesicular venous plexus to the female reproductive tract, parasite eggs can be deposited in the uterus, fallopian tubes, ovaries and other reproductive tissues [19]. This condition is known as female genital schistosomiasis (FGS). FGS can be diagnosed in the lower genital tract through colposcopy, molecular methods, or visualization of parasite eggs in tissue (through biopsy or cytology). The presence of parasite eggs in female genital tissue often leads to an inflammatory response, characterized by the infiltration of immune cells (lymphocytes, neutrophils, eosinophils, etc.) and granuloma formation [20, 21]. Granuloma formation during genital *S. haematobium* infection may ultimately progress to fibrosis and scarring [16]. FGS has been associated with poor reproductive health outcomes including ectopic pregnancy [22] and infertility [23].

Cervical cancer and *S. haematobium* eggs have been reported together in histopathology specimens of women with cervical cancer with detection ranging from 0 – 32% in five countries [21, 22, 24–26]. Most parasite eggs are deposited in the subepithelial tissues [21, 27]. FGS is associated with characteristic changes in the cervical mucosa known as sandy patches, abnormal blood vessels, and rubbery papules [19]. HPV establishes infection in the cervical basal cells and FGS-related damage of the genital epithelium may thus predispose to HPV acquisition and associated inflammation may facilitate persistence of HPV infection. High-risk HPV persistence and *S. haematobium* infection are both individually characterized by modulations in the cervicovaginal immune environment [28, 29]. The Th2 biased cervicovaginal immune environment observed in higher burden FGS and soil-transmitted helminth infection may impair the mechanisms needed for clearance of HPV infection [30, 31]. Such observations suggest that FGS may be a co-factor in HR-HPV persistence and the development of cervical pre-cancer, but the evidence base for this association has not been systematically explored [32, 33]. The aim of this review is to evaluate the association of FGS and high risk (HR)-HPV prevalence, incidence or persistence, cervical pre-cancer (high-grade cervical intraepithelial neoplasia (CIN), or CIN2/3) prevalence and incidence and cervical cancer incidence.

## Methods

### Search strategy and selection criteria

For this systematic review, we searched OvidSP MEDLINE, OvidSP Embase, Global Index Medicus, PubMed and the Wiley Cochrane library complete databases of systematic reviews up to April 20, 2024 with keywords including female genital schistosomiasis, human papillomavirus, cervical intraepithelial neoplasia (CIN), and cervical cancer (Supplement A for search strategy). All abstracts were uploaded into Covidence [34] and screened by two authors (AS and HK). Full-text manuscripts were obtained and an independent full-text review of studies from the consensus list was performed by two authors (AS and HK). The data extractors reached consensus regarding the relevance of the included studies through detailed discussions [34]. Visual-FGS was defined as FGS diagnosed primarily by traditional or hand-held colposcopy. Molecular-FGS was defined as FGS diagnosed primarily by polymerase chain reaction (PCR). Composite-FGS was defined as FGS diagnosed by a combination of methods (histopathology or cytology, colposcopy, and molecular methods).

### Inclusion criteria

Study eligibility was defined on pre-defined exposure and outcome criteria [35]. Prospective cohort, cross-sectional or case–control studies were eligible if they reported an association of the exposure (FGS) with any of the following outcomes: prevalence, incidence or persistence of cervical HR-HPV [14], prevalence or incidence of cervical pre-cancer or incidence of cervical cancer. Longitudinal studies were eligible if they evaluated the association of FGS at enrolment with HR-HPV incidence or persistence or the incidence of cervical pre-cancer or cancer. There were no restrictions on age, language, geographic region, or publication date.

### Data analysis

Data were extracted by one author (AS) and verified by a second author (HK) using a standardized form. Where available, odds ratios (OR) and 95% confidence intervals (CI) were extracted. Due to limited availability of data, no meta-analyses were performed.

### Assessment of study quality

We adapted the Newcastle–Ottawa scale to assess the methodological quality of the studies (Supplement B) and assessment was done by two authors (AS and HK) [36]. Cohort studies were assessed on participant selection (representativeness of the exposed cohort, selection of the exposed cohort, ascertainment of exposure, confirmation the outcome was not present at baseline), comparability of the exposed and non-exposed cohort (including adjusting for age and HIV status in multivariate analysis), and study outcome (assessment of outcome, adequate duration of follow-up, and adequacy of follow-up). Cross-sectional studies were assessed on similar criteria with minor variations (Supplement B).

This article is reported based on the Preferred Reporting Items for Systematic Reviews and Meta-analysis (PRISMA) guidelines [37]. This systematic review is registered on the PROSPERO database at the Centre of Reviews and Dissemination (University of York, York, UK), CRD42023389301 [35].

## Results

**Fig. 1:**
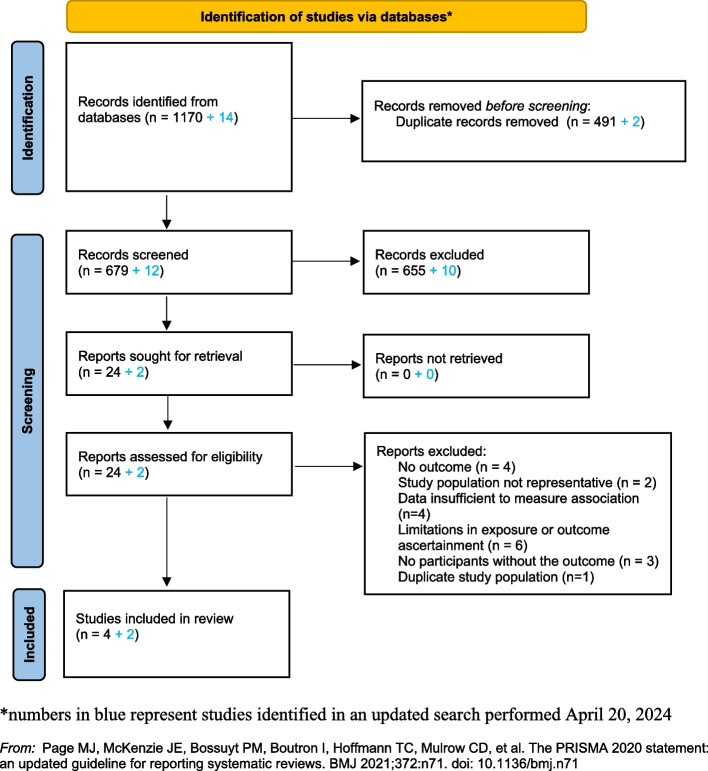
PRISMA 2020 flow diagram for systematic reviews which included databases searches

The search identified 1,170 publications (Fig. 1), among which six articles reported the association of FGS with HR-HPV infection or cervical pre-cancer among 1,081 women with median age ranging from 19 to 32 years (Table 1). The age range of all enrolled participants was 15 – 47 years. All studies were conducted in sub-Saharan Africa in *S. haematobium* endemic settings and published between 1996 and 2023 (Table 1). Five studies were cross-sectional [32, 38–41] and one was a prospective cohort study [33]. Two studies enrolled women from a community setting [32, 33] and four studies recruited women and girls from either schools or clinics [38–41].

**Table 1:** Summary of six studies reporting an association between FGS and HPV, or cervical pre-cancer, or cervical cancer

Author	Location	Study Period	Study Design	Number enrolled	Median Age (years), IQR, Range	FGS definition (method)	Outcome definition (method)	FGS prevalence	Outcome prevalence	Association of FGS and Outcome
**Human Papillomavirus**										
Kjetland 2010 [23, 33]	Zimbabwe	1998–1999	Cohort	*n* = 37	Not Given; 20–49	Cytology, CVL PCR, HP, Colposcopy^a^	HPV persistence (CVL, GP 5 + /6 + PCR; 18 HR subtypes)^b^	53.2% (17/32)	46% (17/37) any 52% (9/17) same subtype 71% (12/17) new type	28.6% (6/21) FGS 18.8% (3/16) FGS negative
Kjetland 2010 [23, 33]	Zimbabwe	1998–1999	Cohort	*n* = 37	Not Given; 20–49	See above	HR-HPV prevalence (CVL, GP 5 + /6 + PCR; 18 HR subtypes)^b^	53.2% (17/32)	22% (54/236)^d^	FGS associated with HR-HPV (baseline) (aOR 1.9, 95% CI 1.1 – 3.6, *p* = 0.03) “neither baseline nor current HYSP were associated with new or remaining HR-HPV (separately or together)”
Kjetland 2010 [23, 33]	Zimbabwe	1998–1999	Cohort	*n* = 37	Not Given; 20–49	See above	Any HPV prevalence (CVL, GP 5 + /6 + PCR; 18 HR subtypes)^b^	53.2% (17/32)	46% (17/37)	FGS associated with any HPV^c^ type (crude OR = 1.6; 95%CI: 0.95–2.8, *p* = 0.08),
Kutz 2023 [41]	Madagascar	2020	Cross-Sectional	*n* = 302	31 (25–37); 18–49	Colposcopy	Any HPV prevalence (CVL)	62.6% (189/302)	42.7% (129/302)	FGS associated with any HPV (0.82 – 1.2) FGS + /HPV + 62.0% FGS + /HPV– 63.0%
Shukla 2023 [40]	South Africa	2011–2013	Cross-sectional	*n* = 933	18.7 (SD 1.6)^e^; 16–22	Colposcopy	Any HPV prevalence (CVL)	22.5% (210/933)	27.0% (149/551)	FGS associated with any HPV aOR 1.71 (1.14 – 2.56), *p* = 0.01
**Cervical Precancer**										
Kjetland 1996 [38]	Malawi	1994	Cross-sectional	*n* = 51	22 (15–47)	HP	Cervical pre-cancer (Histopathology & Cytology – method not specified); Outcome – “dysplasia”	64.7% (33/51)	3.9% (2/51)^d^	3.0% (1/33) FGS^~^ 6.0% (1/18) FGS negative^g^
Pillay 2016 [39]	South Africa	2010–2012	Cross-sectional	*n* = 394	19 (SD 1.64)^e^;	Cytology, CVL PCR	Cervical pre-cancer (Cytology – Bethesda system); Outcome – SCA	2.0% (8/394 by cytology) 9.6% (38/394 by PCR)	17.0% (67/394) LSIL 1.5% (6/394) HSIL 27.1% (107/394) Any SCA	“SCA was not associated with any tests for schistosomiasis used in this study”
Kjetland 2010 [23, 33]	Zimbabwe	1998–1999	Cohort	*n* = 37	Not Given; 20–49	Cytology, CVL PCR, HP, Colposcopy^a^	Cervical pre-cancer (Cytology – CIN I—III); Outcome – squamous intraepithelial dysplasia or HSIL	53.2% 17/32	85.7% (6/7) – women with FGS	HYSP (baseline) and squamous intraepithelial dysplasia (*p* = 0.078) HYSP (follow-up) and squamous intraepithelial dysplasia (aOR 4.8, 95% CI 0.4 – 53) HSIL in FGS (aOR 7.1, 95% CI [0.5 – 92.1], *p* = 0.1)
Rafferty 2021 [32]	Zambia	2018	Cross-sectional	*n* = 237	24 (22–27); 18–31	Genital PCR	Cervical pre-cancer (VIA); Outcome – acetowhite changes	5.9% (14/237)	10.1% (24/237)	Positive VIA and PCR-FGS aOR 6.08 (95% CI: 1.58 – 23.37)
**Squamous Cell Carcinoma**										
Kjetland 1996 [38]	Malawi	1994	Cross-sectional	*n* = 51	22 (15–47); not given	HP^1^	SCC (HP)	64.7% (33/51)	1.9% (1/51)	3.0% (1/33) FGS^f^ 0% (0/18) FGS negative

*aOR* adjusted Odds Ratio, *CVL* Cervicovaginal lavage, *FGS* Female genital schistosomiasis, *HP* Histopathology, *HPV* Human papillomavirus, HYSP Homogeneous yellow sandy patch, *IF* Immunofluorescence, *LSIL* Low grade squamous intraepithelial lesion, HSIL High grade squamous intraepithelial lesion, *NS* Not significant, *PCR* Polymerase chain reaction, *SCA* Squamous cell atypia, *SCC* Squamous cell carcinoma, *SIM* Squamous intraepithelial malignancy, *SD* Standard deviation, VIA Visual inspection with acetic acid

^a^proportions of positive for each method are not reported

^b^high risk subtypes: 16, 18, 26, 31, 33, 35, 39, 45, 51, 52, 53, 56, 58, 59, 66, 68, 73, and 82

^c^data extracted from baseline study (Kjetland EF, Ndhlovu PD, Mduluza T, Gomo E, Gwanzura L, Mason PR, et al. Simple clinical manifestations of genital Schistosoma haematobium infection in rural Zimbabwean women. Am J Trop Med Hyg. 2005;72(3):311–9.)

^d^unclear if baseline or longitudinal data

^e^Denotes mean age

^f^OR not calculated given missing data or small numbers: cervical pre-cancer outcome, OR could be calculated but not felt to be clinically meaningful given small numbers; squamous cell carcinoma, OR could not be calculated due to missing data – 0 cases of squamous cell carcinoma in the FGS negative group

^g^calculated from the text

FGS was diagnosed using molecular methods in two studies [32, 39], biopsy and histopathology in one study [38], colposcopy in two studies [40, 41] and composite methods in one study (primarily colposcopy) [33]. The prevalence of FGS ranged from 5.9% (using molecular methods) [32] to 62.6% (using colposcopy) [41].

Four studies evaluated the association of FGS and cervical pre-cancerous lesions (two reported cytology-based [33, 39], one reported histology-based [38] and one reported visual inspection with acetic acid (VIA)-based outcomes [32]). One study evaluated the association of FGS and HR-HPV prevalence [23]; two evaluated association with any HPV type prevalence [40, 41] and one prospective study evaluated the association of baseline FGS and HR-HPV re-detection over 5 years [33].

### Assessment of study quality

The included studies were assessed for three key criteria: cohort selection, comparability of the cohorts, and ascertainment of exposure and outcome (Table 2; Supplement B). Four included studies were graded as moderate quality [32, 38, 40, 41] based on cohort selection and comparability criteria, often with higher scoring studies adjusting for confounding. Two included studies were graded low to moderate quality [33, 39] based on limitations in exposure and outcome ascertainment and lack of adjustment for confounding when evaluating the association of FGS with HPV or cervical pre-cancer outcomes (Supplement B).

**Table 2:** Risk of bias assessment of cohort studies evaluating the association of FGS and human papillomavirus and cervical precancer using a modified Newcastle Ottawa Scoring system

Study	Selection				Comparability	Outcome			Total **Max 10 stars**	Quality (1–3 low; 4–7 mod; 8–10 high)
	Representativeness of the exposed **Max 1 star**	Selection of the non-exposed **Max 1 star**	Ascertainment of exposure **Max 1 star**	Outcome not present at study onset **Max 1 star**	Adjustment for confounders **Max 2 stars**	Assessment of outcome **Max 1 star**	Duration of follow-up **Max 1 star**	Adequacy of follow-up **Max 1 star**		
Kjetland 2010 – Any HPV Persistence	1 star	1 star	0 stars	0 stars	0 stars	0 stars	0 stars	0 stars	2 stars	Low
Kjetland 2010 – Cervical Pre-cancer	1 star	1 star	0 stars	0 stars	1 star	0 star	1 star	1 star	5 stars	Moderate

Strengths of the included studies were that all six studies were from *S. haematobium* endemic areas and the majority were representative of the population of women living with FGS. A limitation was that studies employed heterogeneous methods to ascertain a FGS diagnosis, with only half of the included studies primarily using high-specificity (histopathology or molecular) methods [32, 38, 39]. Another limitation was in outcome assessment;. Of the four studies that assessed cervical pre-cancer, only one used histology [38]. Two studies used cytology alone [33, 39] and one used VIA [32]. Additionally, only one of the included studies performed a power calculation [40], so the majority of studies were not powered to detect a difference between the outcome and the exposure. In some studies the sample sizes were small, making the effect sizes difficult to interpret.

#### Association of FGS and HPV

One study reported the association of FGS and HR-HPV prevalence at baseline and type-specific persistence over 5 years follow-up (Table 2) [33]. Among 236 women in Zimbabwe (age range 15–49 years), there was evidence that a composite FGS diagnosis (eggs in cytology, cervicovaginal lavage (CVL) PCR, biopsy, and colposcopy, with the majority using colposcopy) was associated with HR-HPV prevalence at baseline (adjusted OR (aOR) = 1.9, 95% CI:1.1–3.6, *p* = 0.03, adjusted for age). Among 37 women who were HPV positive at enrolment and followed over 5 years, HPV type persistence was higher in women with FGS detection at any timepoint (composite definition) compared to those without FGS (28.6% [6/21] vs. 18.8% [3/16], respectively (effect estimate not reported)) [33]. There was weak evidence of an association between any HPV type and FGS (crude OR = 1.6; 95%CI: 0.95–2.8, *p* = 0.08), however it is unclear whether this association uses baseline or longitudinal data [33].

Two cross-sectional studies reported the association of FGS with any HPV type prevalence with conflicting results. Among 302 women in Madagascar, there was no evidence that visual-FGS (colposcopy defined) was associated with any HPV (OR = 1.0, 95%CI: 0.82–1.2) [41]. In contrast, there was strong evidence that visual-FGS (colposcopy defined) was associated with any HPV (aOR = 1.71, 95%CI:1.14–2.56], *p* = 0.01) among 933 women in KwaZulu-Natal [40]. The key differences between the studies in Madagascar [41] and KwaZulu-Natal [40] were median age (31 [25–37]) vs. mean age 18.2 [standard deviation 1.6]), FGS prevalence (62.6% vs 22.5%), and HPV detection (42.7% [using CVL] vs 22.5% [using CVL], respectively.

#### Association of FGS with cervical pre-cancer and cancer

Four studies evaluated the association between FGS and cervical pre-cancer (Table 1) [32, 33, 38, 39]. Among 237 women in Zambia with a median age of 24 years, [32], there was strong evidence of an association of molecular-FGS (*Schistosoma* DNA by PCR in any of CVL, vaginal swab or cervical swab with abnormal VIA exam (aOR = 6.08, 95%CI: 1.58–23.37, adjusted for age and HIV status)) [32]. The study reporting histology outcomes provided the proportion of participants with cervical pre-cancer [38]. Of the two studies that reported cytology outcomes, one did not find an association between any schistosomiasis diagnostics and cervical pre-cancer [39], and one did not find an association between visual-FGS and cervical dysplasia (aOR 4.8, 95% CI 0.4 – 53) [33] (Table 1). The longitudinal study from Zimbabwe reported strong evidence of an association between FGS (eggs in Pap smear) and “clinical suspicion of malignancy” in the baseline univariate analysis (OR 3.21, 95% CI 1.03 – 10.07, *p* < 0.045), but this association was not seen in multivariate analysis (effect estimate not reported) [42].

Of the six included studies, no studies evaluated the association between FGS and cervical cancer. However, one cross sectional study among 51 women in Malawi [38] reported one case of histology-verified squamous cell carcinoma (SCC) in a participant with FGS, detected in the cervical biopsy.

## Discussion

In this review we report that there is limited evidence, from a small number of studies, of an association between visual or molecular FGS and HR-HPV prevalence and cervical pre-cancer [32, 33, 40]. However there was inconsistency in the reported associations, which may be attributed to the heterogeneity in ascertainment of both the exposure (FGS) and the outcome (cervical pre-cancer).

A challenge across many of the studies was the ascertainment of the exposure (FGS) and outcome (HPV and cervical pre-cancer) measures. In the six studies included in this systematic review, there were variations in the reported FGS prevalence (Table 2). One explanation could be the difference in *S. haematobium* prevalence across study sites. In FGS diagnostics, there is no agreed reference standard that is field friendly, widely available, scalable and optimizes both sensitivity and specificity [19]. For example, molecular methods, such as *Schistosoma* PCR have imperfect sensitivity (80.0%) but excellent specificity (100.0%), while detection of mucosal FGS lesions (sandy patches) has imperfect sensitivity (81.6%) and poor specificity (42.4%) [43, 44]. Another challenge posed by the use of low-specificity FGS diagnostics is the limited agreement between reviewers of visual-FGS cases [45]. Performing colposcopy for the evaluation of visual-FGS requires rigorous adherence to quality standards [46]. Ultimately, however, the interpretation of the photocolposcopic images is subjective and can vary by reader, their visual acuity, and their degree of training. Data from Zambia suggest there is a low level of correlation between expert reviewers when diagnosing FGS based on photocolposcopic images [45]. However, molecular-FGS detects DNA from *S. haematobium* eggs, is not-subjective, and is highly reproducible. Thus, a substantial concern with employing low specificity diagnostics is the possibility of false positive results. FGS diagnosis in the community should be guided by the availability of diagnostics and clinical experience. However, to ensure accurate FGS case ascertainment in research settings, detection methods with high specificity, such as histopathology and molecular methods, are warranted.

Similarly, the gold standard measurement for cervical pre-cancer is histology verified cervical intraepithelial neoplasia, grades 2 or 3, which requires a tissue biopsy for histopathology verification. Cervical cytology and VIA are widely used in cervical cancer screening programmes but are imperfect measures of cervical pre-cancer due to the variable sensitivity reported [47], linked to operator variability and training. Most of the studies included in our review used cytological or VIA defined outcomes for cervical pre-cancer, rather than histology, reflecting the difficulties and ethical implications associated with cervical biopsy taking as part of research studies, in particular given the younger age range of the women included in the studies within this review.

Of the four studies that evaluated cervical pre-cancer outcomes, one study used histology [38], one study used VIA [32] and two used cervical cytology to define cervical pre-cancer. Although the studies employed cytology and histology, the authors used different terminologies and thresholds to define cervical pre-cancer, making it challenging to verify if low or high grade lesions were identified. Of these three studies, the outcomes reported were: “dysplasia” [38], “squamous cell atypia” [39], and both “high grade squamous intraepithelial lesion” and “squamous intraepithelial dysplasia” [33] (Table 1). None of these three studies showed strong evidence of an association between FGS and cervical pre-cancer [33, 38, 39]. Thus, heterogeneity in defining cervical pre-cancer limits confidence in the findings. Only one study showed strong evidence of an association between molecular FGS and cervical acetowhite changes, identified by VIA exam in Zambia [32]. Since the sensitivity and specificity of VIA are limited, its use can yield both false negative and false positive results. Thus, despite the strong evidence of an association between molecular-FGS and a VIA-based diagnosis of cervical pre-cancer, it is challenging to conclusively accept or reject an association using VIA-based testing alone [32]. Due to the biological plausibility of an association between FGS and cervical pre-cancer, further research is needed and work evaluating the association between molecular-FGS and cervical pre-cancer using DNA-based HPV testing is ongoing in Zambia [48].

Molecular testing using HPV-DNA based tests is a reproducible and reliable measure for HPV infection. The associations reported between FGS and HPV outcomes were mixed, although there was also heterogeneity in outcome definitions, with some studies reporting associations with any HPV type and others any HR-HPV type. However only one longitudinal study reported an increased risk of any HPV persistence in women with baseline FGS compared to those without over 5 years. It should be noted, however that a 5 year period may be too long to define type-specific persistence as prospective studies of incident and prevalent infections among young women have shown that up to half of infections clear within 6 months and approximately 90% clear within 2 years after acquisition [49, 50]. Additionally, women could acquire a new infection in the 5 year time period. Based on the available data with heterogeneity in outcome and exposure ascertainment, it is not possible to conclude or exclude an association between FGS and HPV until high-specficity methods are used across studies.

The association between HR-HPV and cervical cancer is well established, as is the association between *S. haematobium* and squamous cell carcinoma of the bladder. *S. haematobium* is classified by the International Agency for Research on Cancer (IARC) as a group 1 carcinogen, or “carcinogenic to humans” [13, 14]. Our review found no studies evaluating the association of FGS and cervical cancer. However, one small cross-sectional study reported histology-verified FGS in biopsy tissue in one woman with cervical squamous cell carcinoma [38].

Oncogenic human papillomavirus is necessary for the development of the majority of invasive cervical cancer [2, 51], and is an independent risk factor for cervical pre-cancer [2, 51]. HPV infects the undifferentiated, proliferating basal cells of the cervicovaginal epithelium [52]. HPV gains access to the basal cells as a consequence of microabrasion, resulting from co-existence of other sexually transmitted infections or other co-factors that may compromise the epithelial barrier including hormonal contraception [53]. Genital schistosomiasis is associated with epithelial damage [54], and is manifest by the development of characteristic cervicovaginal mucosal findings, such as sandy patches and rubbery papules [19]. FGS-related epithelial disruption may thus predispose the basal cells of the cervical epithelium to HPV infection. In a study among Tanzanian women with urinary *S. haematobium* infection, cervicovaginal gene expression analysis revealed altered expression of matrix metalloproteinases (MMPs) in the cervical mucosa during *S. haematobium* infection [55]. MMPs regulate the breakdown of extracellular matrix proteins and if altered MMP expression in *S. haematobium* infection is associated with a compromised epithelial barrier, this may allow HPV entry. Further research is required to evaluate if *S. haematobium* induced changes in the structural integrity of the cervicovaginal epithelium might facilitate HPV acquisition.

Our understanding of the pathophysiology supporting the association between *S. haematobium* and squamous cell cancer of the bladder is evolving, with much of the understanding of the mechanism between *S. haematobium* infection and bladder cancer derived from animal models [56, 57]. While the presence of adult *S. haematobium* worms in the systemic vasculature may alter the systemic immune milieu, local lodging of eggs in bladder tissue has localized effects on the genitourinary immune environment [58]. A Th2 cytokine response has been described in the systemic circulation and associated pelvic lymph nodes following *S. haematobium* egg deposition [56]. It may be that *S. haematobium* associated transition to a Th2-biased immune environment is associated with dysregulation of HPV control, potentially promoting oncogenesis through HPV persistence. Women who clear HPV infection are more likely to have detectable levels of IFN-γ, indicative of a Th1 immune response compared to those whose infections persist [59] and a Th2 response (IL-4 and IL-10) may be more likely among those whose HPV infections progress to CIN3 [60]. FGS has likewise been associated with alterations in the cervicovaginal immune environment, with a shift towards a Th2 and pro-inflammatory profile in high burden infections [61]. In a murine model of helminth and viral co-infection, the Th2 biased environment after acute *S. mansoni* infection was associated with reactivation of latent herpesviruses [62]. This suggests that helminth and viral co-infections may exist in a counterpoise regulated by the effect of cytokines on viral replication and control [63, 64]. It is a priority in future research to evaluate the impact of *S. haematobium* and HPV co-infection on HPV incidence and persistence. Further research is needed to evaluate whether *S. haematobium* infection, and subsequent FGS, might promote an immunologic environment which facilitates HPV persistence.

This manuscript is the first systematic review to evaluate the association of FGS and HPV infection, cervical pre-cancer, and cervical cancer. However, there are limitations to consider. First, the body of literature that evaluates the association between FGS and HPV, cervical pre-cancer, and cervical cancer is remarkably limited. Schistosomiasis is a neglected tropical disease and data regarding gender-specific manifestations, such as female genital schistosomiasis, are limited with fewer than 15,000 cases adequately described [19]. Second, among the included studies, there were challenges in accurate exposure and outcome ascertainment. This makes it challenging to confidently confirm the presence or absence of an association between FGS and HPV infection, cervical pre-cancer and cervical cancer. This systematic review has identified a number of knowledge gaps: first, limited publications evaluate the pathophysiology of human FGS, HPV, and cervical pre-cancer. Second, there are gaps in our understanding of the natural history of FGS and HPV coinfection. Third, the epidemiologic association between FGS, HPV and cervical pre-cancer requires further exploration in well-powered studies with high specificity diagnostics.

## Conclusion

In line with the WHO goal to eliminate cervical cancer as a public health problem [47] the number of countries with HPV vaccination and cervical cancer screening and treatment programmes is increasing [8, 9]. Such programmes offer opportunity to provide a package of care for women at risk of cervical cancer, including integrated care for FGS and sexually transmitted infections. However, robust evidence on the dual burden and association of FGS and cervical pre-cancer is needed to mobilize funding and motivate stakeholders to integrate FGS care into existing cervical cancer screening programmes.

WHO currently recommends using molecular methods (HPV DNA detection) as the primary cervical screening test rather than VIA or cytology in screening and treatment approaches among both the general population of women and women living with HIV [47]. Future research on the association between FGS and HPV, cervical pre-cancer, and cancer should prioritize high specificity FGS diagnostic methods (molecular methods and histology-verified outcomes) over evaluations of FGS by lower specificity methods (visual imaging). Our findings highlight the knowledge gaps in the association between FGS, HPV, and cervical pre-cancer and are a call to action for future investigations in this neglected field.

## Supplementary Information

Supplementary Material 1.

Supplementary Material 2.

## Data Availability

No datasets were generated or analysed during the current study.

## Abbreviations

APC: Antigen presenting cell
aOR: Adjusted odds ratio
CI: Confidence interval
CIN: Cervical intraepithelial neoplasia
DNA: Deoxyribonucleic acid
FGS: Female genital schistosomiasis
HPV: Human papillomavirus
HR: High risk
IARC: International Agency for the Research on Cancer
IL: Interleukin
mRNA: Messenger ribosomal nucleic acid
MMP: Matrix metalloproteinases
OR: Odds ratio
PCR: Polymerase chain reaction
SCC: Squamous cell carcinoma
Th2: T helper 2
VIA: Visual inspection with acetic acid
