# Supplementary material for: Association of female genital schistosomiasis and human papillomavirus and cervical pre-cancer: a systematic review
Source: BMC Womens Health. 2025 Jan 3;25:2. doi: 10.1186/s12905-024-03514-0 (PMC11697648; doi:10.1186/s12905-024-03514-0)
Supplement: Supplementary file 1 — Supplementary Material 1. [file 12905_2024_3514_MOESM1_ESM.docx]

**Appendix A - Search Strategy**

## Databases

The following databases were searched on March 16, 2023

- Global Health

The following databases were searched on April 5, 2023

- OvidSP Medline
- OvidSP Embase
- Wiley Cochrane Library, complete databases
- Pubmed
- Global Index Medicus

## Information management

All citations identified by our searches were imported into EndNote X9 software. Duplicates were identified and removed using the method described on the LSHTM Library and Archive Services blog.^[[1]](#footnote-1)^

# Results

A total of 1170 results were retrieved by the search, 491 were identified as duplicates. Number of results are listed in the table below.

| Database name | **Total number of results** |
| --- | --- |
| OvidSP Medline | 158 |
| OvidSP Embase | 381 |
| Wiley Cochrane Library | Cochrane Database of Systematic Reviews: 0 Cochrane Database of Systematic Reviews protocols: 0  Cochrane Central Register of Controlled Trials: 2 Cochrane Clinical Answers:0 |
| OvidSP Global Health | 199 |
| PubMed | 252 |
| Global Index Medicus | 178 |
| **Total** | **1170** |

# Appendix: Search strategies

This appendix provides full details of all search strings used for bibliographic databases, with dates and number of references returned and notes explaining any unusual search techniques or syntax. EndNote X9 referencing system was used. The original search was conducted on April 5, 2023. The EndNote X9 import order is provided, as the deduplication technique keeps the first uploaded copy of the reference by default. In all searches, numbers in right columns for each row show the number of hits retrieved.

**Ovid MEDLINE**

| Database name | Medline |
| --- | --- |
| Database platform | OvidSP |
| Dates of database coverage | Ovid MEDLINE(R) 1946 to March week 4 2023 |
| Date searched | 05 April 2023 |
| Searched by | AS |
| Number of results | 158 |
| Search strategy notes | Search lines ending in a ‘/’ are subject heading searches. Search lines beginning ‘exp’ are exploded subject heading searches. Search lines ending in .ti,ab. search in the title and abstract only. or/*x-y* combines search sets in the range *x-y* with Boolean operator OR. * is used for truncation of words. adj*n* searches for words within *n* words of each other. |

| 1 | Schistosoma haematobium.mp or exp Schistosoma haematobium | 3531 |
| --- | --- | --- |
| 2 | Schistosomiasis.mp or exp schistosomiasis/ | 27514 |
| 3 | Schistosoma.mp or exp Schistosoma/ | 23263 |
| 4 | Bilharz*.mp | 3040 |
| 5 | 1 or 2 or 3 or 4 | 34595 |
| 6 | Female genital.mp or exp Genitalia, female/ | 277822 |
| 7 | Female genital system.mp | 318 |
| 8 | Female genital tract.mp | 4585 |
| 9 | cervic*.mp | 276559 |
| 10 | cervix.mp. or exp Cervix Uteri/ | 68847 |
| 11 | Cervico*.mp. | 15548 |
| 12 | 6 or 7 or 8 or 9 or 10 or 11 (female genital or cervix) | 534482 |
| 13 | 5 and 12 (female genital sh) | 473 |
| 14 | Genital Neoplasms, Female/ or genital neoplasm female.mp | 253901 |
| 15 | Cervical intraepithelial neoplasia.mp or exp Uterine Cervical Dysplasia | 16953 |
| 16 | cancer.mp. or exp Neoplasms/ | 4071472 |
| 17 | Exp uterine cervical diseases/ | 92921 |
| 18 | premalignant.mp. | 10737 |
| 19 | neoplas*.mp. | 3283044 |
| 20 | dysplas*.mp. | 103693 |
| 21 | Exp Uterine Cervical Neoplasms/ or dyskaryosis.mp | 84404 |
| 22 | Squamous*.mp. | 195242 |
| 23 | Cancer.mp or exp Neoplasms/ | 4084662 |
| 24 | Cancerous.mp | 24058 |
| 25 | Exp Carcinoma, Squamous cell/ or exp Carcinoma/ or carcinoma.mp or exp Carcinoma, Adenosquamous/ | 1004671 |
| 26 | CIN*.mp. | 162913 |
| 27 | CIN1*.mp. | 918 |
| 28 | CINI*.mp. | 692 |
| 29 | CIN2*.mp. | 2280 |
| 30 | CINII*.mp. | 214 |
| 31 | CIN3*.mp. | 1460 |
| 32 | CINIII*.mp. | 77 |
| 33 | Exp Uterine Cervical Diseases/ | 92921 |
| 34 | Pre-cancer* | 1723 |
| 35 | 14 or 15 or 16 or 17 or 18 or 19 or 20 or 21 or 22 or 23 or 24 or 25 or 26 or 27 or 28 or 29 or 30 or 31 or 32 or 33 or 34 [cervical precancer or cancer] | 4389640 |
| 36 | HPV.mp. or exp Papillomavirus Infections/ or exp Papillomaviridae/ | 64201 |
|  |  |  |
| 37 | human papillomavirus.mp. or exp Alphapapillomavirus/ | 40642 |
| 38 | 36 or 37 [hpv] | 67495 |
| 39 | 35 or 38 [hpv or cervical precancer/cancer) | 4408589 |
| 40 | 13 and 39 (fgs and [hpv or cervical precancer/cancer]) | 158 |
|  |  |  |
|  |  |  |
|  |  |  |

**Ovid EMBASE**

| Database name | OVID EMBASE |
| --- | --- |
| Dates of database coverage | 1916 to 2023 Week 13 |
| Date searched | 5 April 2023 |
| Searched by | AS |
| Number of results | 381 |
| Search strategy notes | Search lines ending in a ‘/’ are subject heading searches. Search lines beginning ‘exp’ are exploded subject heading searches. Search lines ending in .ti,ab. search in the title and abstract only. or/*x-y* combines search sets in the range *x-y* with Boolean operator OR. * is used for truncation of words. adj*n* searches for words within *n* words of each other. |

| 1 | ‘Schistosoma haematobium’ or exp Schistosoma haematobium | 5827 |
| --- | --- | --- |
| 2 | ‘Schistosomiasis’ | 36477 |
| 3 | ‘Schistosoma’ | 32039 |
| 4 | Bilharz* | 6691 |
| 5 | 1 or 2 or 3 or 4 | 50,491 |
| 6 | ‘Female genital system’ | 114471 |
| 7 | ‘female genital’ | 156,628 |
| 8 | ‘female genital tract’ | 39,577 |
| 9 | Cervic* | 438,567 |
| 10 | ‘uterine cervix’ | 202,218 |
| 11 | Cervico* | 41628 |
| 12 | #6 OR #7 OR #8 OR #9 OR #10 OR #11 (female genital or cervix) | 641,371 |
| 13 | #5 and #12 (female genital sh) | 680 |
| 14 | ‘genital tract tumor’ | 8626 |
| 15 | ‘uterine cervix dysplasia’ OR ‘uterine cervix disease’ OR ‘cervical’ | 407,777 |
| 16 | ‘cervical intraepithelial neoplasia’ OR ‘uterine cervix tumor’ | 36,969 |
| 17 | ‘precancer*’ | 40.728 |
| 18 | ‘premalignant’ | 20,627 |
| 19 | neoplas* or ‘neoplasm’ | 1,470,950 |
| 20 | Dysplas* or ‘dysplasia’ | 180,900 |
| 21 | ‘dyskaryosis’ | 877 |
| 22 | Squamous* | 301,006 |
| 23 | ‘cancer’ | 5,205,021 |
| 24 | ‘cancerous’ | 41,246 |
| 25 | ‘squamous cell carcinoma’ OR ‘adenosquamous carcinoma’ OR ‘carcinoma’ | 1,454,701 |
| 26 | CIN* | 550,115 |
| 27 | CIN1* | 1822 |
| 28 | CINI* | 7209 |
| 29 | CIN2* | 4270 |
| 30 | CINII* | 847 |
| 31 | CIN3* | 2596 |
| 32 | CINIII* | 230 |
| 33 | (‘Cervical intraepithelial neoplasia’ or cervical) AND Intraepithelial AND Neoplasia | 13856 |
| 34 | Pre-cancer* | 3625 |
| 35 | #14 OR #15 OR #16 OR #17 OR #18 OR #19 OR #20 OR #21 OR #22 OR #23 OR #24 OR #25 OR #26 OR #27 OR #28 OR #29 OR #30 OR #31 OR #32 OR #33 OR #34 | 6,845,463 |
| 36 | HPV OR ‘papillomavirus’ OR ‘papillomavirus infection’ | 82039 |
| 37 | human AND papillomavirus | 66538 |
| 38 | 36 or 37 (hpv) | 91,353 |
| 39 | 35 or 38 (HPV or cervical precancer/cancer) | 4,408,589 |
| 40 | 13 and 39 (FGS and [HPV or cervical precancer/cancer]) | 381 |

|  |  |  |
| --- | --- | --- |

**Global Health**

| Database name | Global Health |
| --- | --- |
| Dates of database coverage | 1910 to 2023 Week 10 |
| Date searched | 16 March 2023 |
| Searched by | HK |
| Number of results | 199 |
| Search strategy notes | Search lines ending in a ‘/’ are subject heading searches. Search lines beginning ‘exp’ are exploded subject heading searches. Search lines ending in .ti,ab. search in the title and abstract only. or/*x-y* combines search sets in the range *x-y* with Boolean operator OR. * is used for truncation of words. adj*n* searches for words within *n* words of each other. |

| 1 | Schistosoma haematobium.mp. or exp Schistosoma haematobium/ | 8136 |
| --- | --- | --- |
| 2 | Schistosomiasis.mp. or exp schistosomiasis/ | 49257 |
| 3 | Schistosoma.mp. or exp Schistosoma/ | 53361 |
| 4 | Bilharz*.mp. | 48633 |
| 5 | 1 or 2 or 3 or 4 | 53797 |
| 6 | Female genital.mp. or exp Genitalia, female/ | 6836 |
| 7 | Female genital system.mp. [mp=abstract, title, original title, heading words, cabicodes words] | 3562 |
| 8 | Female genital tract.mp. [mp=abstract, title, original title, heading words, cabicodes words] | 899 |
| 9 | cervic*.mp. | 37130 |
| 10 | cervix.mp. or exp Cervix Uteri/ | 23464 |
| 11 | Cervico*.mp. | 2296 |
| 12 | 6 or 7 or 8 or 9 or 10 or 11 | 44357 |
| 13 | 5 and 12 | 392 |
| 14 | Genital Neoplasms, Female/ | 0 |
| 15 | Cervical Intraepithelial Neoplasia/ or exp Uterine Cervical Neoplasms/ | 2139 |
| 16 | Uterine Cervical Dysplasia/ or exp Uterine Cervical Diseases/ or cervical.mp. | 34652 |
| 17 | Precancerous Conditions/ or precancer*.mp. | 2783 |
| 18 | pre-cancer*.mp. | 508 |
| 19 | premalignant.mp. | 1134 |
| 20 | neoplas*.mp. | 288243 |
| 21 | dysplas*.mp. | 5300 |
| 22 | dyskaryosis.mp. | 112 |
| 23 | Squamous*.mp. | 13739 |
| 24 | cancer.mp. or exp Neoplasms/ | 330894 |
| 25 | cancerous.mp. | 3309 |
| 26 | Carcinoma, Squamous Cell/ or carcinoma*.mp. or exp Carcinoma/ or exp Carcinoma, Adenosquamous/ | 65287 |
| 27 | CIN*.mp. | 34669 |
| 28 | CIN1*.mp. | 391 |
| 29 | CINI*.mp. | 206 |
| 30 | CIN2*.mp. | 1275 |
| 31 | CINII*.mp. | 80 |
| 32 | CIN3*.mp. | 789 |
| 33 | CINIII*.mp. | 25 |
| 34 | Cervical intraepithelial neoplasia.mp. or exp Cervical Intraepithelial Neoplasia/ | 3743 |
| 35 | 14 or 15 or 16 or 17 or 18 or 19 or 20 or 21 or 22 or 23 or 24 or 25 or 26 or 27 or 28 or 29 or 30 or 31 or 32 or 33 or 34 | 384906 |
| 36 | HPV.mp. or exp Papillomavirus Infections/ or exp Papillomaviridae/ | 24520 |
| 37 | human papillomavirus.mp. | 19598 |
| 38 | 36 or 37 | 24862 |
| 39 | 35 or 38 | 389883 |
| 40 | 13 and 39 | 199 |
|  |  |  |

**PUBMED**

| Database name | PUBMED |
| --- | --- |
| Dates of database coverage | 1957 to 2023 Week 10 |
| Date searched | 5 April 2023 |
| Searched by | AS |
| Number of results | 252 |
| Search strategy notes | Search lines ending in a ‘/’ are subject heading searches. Search lines beginning ‘exp’ are exploded subject heading searches. Search lines ending in .ti,ab. search in the title and abstract only. or/*x-y* combines search sets in the range *x-y* with Boolean operator OR. * is used for truncation of words. adj*n* searches for words within *n* words of each other. |

| 1 | Schistosoma haematobium or exp Schistosoma haematobium/ | 24,357 |
| --- | --- | --- |
| 2 | Schistosomiasis or exp schistosomiasis/ | 29,399 |
| 3 | Schistosoma or exp Schistosoma/ | 24,357 |
| 4 | Bilharz* | 4,298 |
| 5 | 1 or 2 or 3 or 4 | 37,622 |
| 6 | Female genital or exp Genitalia, female/ | 338,044 |
| 7 | Female genital system | 287,154 |
| 8 | Female genital tract | 292,063 |
| 9 | cervic*.mp. | 316,811 |
| 10 | cervix.mp. or exp Cervix Uteri/ | 72,939 |
| 11 | Cervico*.mp. | 24,043 |
| 12 | 6 or 7 or 8 or 9 or 10 or 11   ((((((Female genital or exp Genitalia, female/) or (Female genital or exp Genitalia, female/)) or (Female genital system)) or (female genital tract)) or (cervic*)) or (cervix or exp cervix uteri)) or (cervico*) | 628,301 |
| 13 | 5 and 12 | 617 |
| 14 | Genital Neoplasms, Female/ | 257,003 |
| 15 | Cervical Intraepithelial Neoplasia/ or exp Uterine Cervical Neoplasms/ | 20,497 |
| 16 | Uterine Cervical Dysplasia/ or exp Uterine Cervical Diseases/ or cervical | 681,729 |
| 17 | Precancerous Conditions/ or precancer* | 72,285 |
| 18 | pre-cancer* | 2,102 |
| 19 | premalignant | 73,100 |
| 20 | neoplas* | 3,337,274 |
| 21 | dysplas* | 113,968 |
| 22 | dyskaryosis | 417 |
| 23 | Squamous* | 218,308 |
| 24 | cancer or exp Neoplasms/ | 4,833,211 |
| 25 | cancerous.mp. | 4,832,276 |
| 26 | Carcinoma, Squamous Cell/ or carcinoma*.mp. or exp Carcinoma/ or exp Carcinoma, Adenosquamous/ | 1,003,267 |
| 27 | CIN*.mp. | 20,928 |
| 28 | CIN1*.mp. | 1110 |
| 29 | CINI*.mp. | 829 |
| 30 | CIN2*.mp. | 2770 |
| 31 | CINII*.mp. | 227 |
| 32 | CIN3*.mp. | 16516 |
| 33 | CINIII*.mp. | 156 |
| 34 | Cervical intraepithelial neoplasia.mp. or exp Cervical Intraepithelial Neoplasia/ | 19944 |
| 35 | 14 or 15 or 16 or 17 or 18 or 19 or 20 or 21 or 22 or 23 or 24 or 25 or 26 or 27 or 28 or 29 or 30 or 31 or 32 or 33 or 34  ((((((((((((((((((((Genital Neoplasms, Female/) or (Cervical Intraepithelial Neoplasia/ or exp Uterine Cervical Neoplasms/)) or (Uterine Cervical Dysplasia/ or exp Uterine Cervical Diseases/ or cervical)) or (Precancerous Conditions/ or precancer*)) or (pre-cancer*)) or (premalignant)) or (neoplas*)) or (dysplas*)) or (dyskaryosis)) or (Squamous*)) or (cancer or exp Neoplasms/)) or (cancerous)) or (Carcinoma, Squamous Cell/ or carcinoma* or exp Carcinoma/ or exp Carcinoma, Adenosquamous/)) or (CIN)) or (CIN1)) or (CINI)) or (CIN2)) or (CINII)) or (CIN3)) or (CINIII*)) or (Cervical intraepithelial neoplasia or exp Cervical Intraepithelial Neoplasia/) | 5,407,272 |
| 36 | HPV.mp. or exp Papillomavirus Infections/ or exp Papillomaviridae/ | 51,835 |
| 37 | human papillomavirus.mp. | 49,767 |
| 38 | 36 or 37 (hpv)  (human papillomavirus) or (HPV or exp Papillomavirus Infections/ or exp Papillomaviridae/) | 62,765 |
| 39 | 35 or 38 (hpv or cervical precancer/cancer)   (((human papillomavirus) or (HPV or exp Papillomavirus Infections/ or exp Papillomaviridae/))) OR (((((((((((((((((((((Genital Neoplasms, Female/) or (Cervical Intraepithelial Neoplasia/ or exp Uterine Cervical Neoplasms/)) or (Uterine Cervical Dysplasia/ or exp Uterine Cervical Diseases/ or cervical)) or (Precancerous Conditions/ or precancer*)) or (pre-cancer*)) or (premalignant)) or (neoplas*)) or (dysplas*)) or (dyskaryosis)) or (Squamous*)) or (cancer or exp Neoplasms/)) or (cancerous)) or (Carcinoma, Squamous Cell/ or carcinoma* or exp Carcinoma/ or exp Carcinoma, Adenosquamous/)) or (CIN)) or (CIN1)) or (CINI)) or (CIN2)) or (CINII)) or (CIN3)) or (CINIII*)) or (Cervical intraepithelial neoplasia or exp Cervical Intraepithelial Neoplasia/)) | 5,418,889 |
| 40 | 13 and 39 (fgs and [hpv or cervical precancer/cancer])  (((human papillomavirus) or (HPV or exp Papillomavirus Infections/ or exp Papillomaviridae/))) OR (((((((((((((((((((((Genital Neoplasms, Female/) or (Cervical Intraepithelial Neoplasia/ or exp Uterine Cervical Neoplasms/)) or (Uterine Cervical Dysplasia/ or exp Uterine Cervical Diseases/ or cervical)) or (Precancerous Conditions/ or precancer*)) or (pre-cancer*)) or (premalignant)) or (neoplas*)) or (dysplas*)) or (dyskaryosis)) or (Squamous*)) or (cancer or exp Neoplasms/)) or (cancerous)) or (Carcinoma, Squamous Cell/ or carcinoma* or exp Carcinoma/ or exp Carcinoma, Adenosquamous/)) or (CIN)) or (CIN1)) or (CINI)) or (CIN2)) or (CINII)) or (CIN3)) or (CINIII*)) or (Cervical intraepithelial neoplasia or exp Cervical Intraepithelial Neoplasia/)) AND ((((bilharz*) or (Schistosoma or exp Schistosoma/)) or (schistosomiasis or exp schistosomiasis/)) or (schistosoma haematobium.mp or exp schistosoma haematobium)) AND (((((((Female genital or exp Genitalia, female/) or (Female genital or exp Genitalia, female/)) or (Female genital system)) or (female genital tract)) or (cervic*)) or (cervix or exp cervix uteri)) or (cervico*)) | 252 |

**Cochrane Databases**

| Database name | Cochrane Databases |
| --- | --- |
| Dates of database coverage | April 1995* |
| Date searched | April 5, 2023 |
| Searched by | AS |
| Number of results | 2 |
| Wiley Cochrane Library | Cochrane Database of Systematic Reviews: 0 Cochrane Database of Systematic Reviews protocols: 0  Cochrane Central Register of Controlled Trials: 2 Cochrane Clinical Answers: 0  Cochrane Editorials: 0 |
| Number of results once duplicates removed | 2 |
| Search strategy notes | Search lines ending in a ‘/’ are subject heading searches. Search lines beginning ‘exp’ are exploded subject heading searches. Search lines ending in .ti,ab. search in the title and abstract only. or/*x-y* combines search sets in the range *x-y* with Boolean operator OR. * is used for truncation of words. adj*n* searches for words within *n* words of each other. |

| ID | Search | Hits |
| --- | --- | --- |
| 1 | (schistosomiasis):ti,ab,kw | 669 |
| 2 | (schistosoma):ti,ab,kw | 437 |
| 3 | (bilharz*):ti,ab,kw | 70 |
| 4 | ("S. haematobium" or "schistosoma haematobium"):ti,ab,kw | 179 |
| 5 | 1 or 2 or 3 or 4  [s haematobium] | 772 |
| 6 | (genitalia, female):ti,ab,kw OR (female genital):ti,ab,kw | 5105 |
| 7 | (female genital tract):ti,ab,kw | 1751 |
| 8 | (female genital system) ti,ab,kw | 1430 |
| 9 | (cervix):ti,ab,kw OR ("uterine cervix"):ti,ab,kw AND ("uterine cervices"):ti,ab,kw | 8976 |
| 10 | (cervic*):ti,ab,kw | 1492 |
| 11 | (cervico*):ti,ab,kw | 796 |
| 12 | #6 or #7 or #8 or #9 or #10 or #11 [female genital or cervix] | 32349 |
| 13 | #5 AND #12 [female genital sh] | 7 |
| 14 | (genital neoplasms, female):ti,ab,kw | 867 |
| 15 | ("cervical intraepithelial neoplasia"):ti,ab,kw OR (uterine cervical neoplasms):ti,ab,kw | 3644 |
| 16 | (uterine cervical dysplasia):ti,ab,kw OR (uterine cervical disease*):ti,ab,kw OR (cervical):ti,ab,kw | 24680 |
| 17 | ("precancerous condition"):ti,ab,kw OR (precancer*):ti,ab,kw | 1434 |
| 18 | ("pre-cancer*"):ti,ab,kw | 193 |
| 19 | ("premalignant"):ti,ab,kw | 545 |
| 20 | (neoplas*):ti,ab,kw | 108094 |
| 21 | (dysplas*):ti,ab,kw | 4924 |
| 22 | (dyskaryosis):ti,ab,kw | 43 |
| 23 | (squamous*):ti,ab,kw | 12267 |
| 24 | ("Cancer"):ti,ab,kw OR (neoplasm):ti,ab,kw | 218933 |
| 25 | (cancerous):ti,ab,kw | 194574 |
| 26 | ("squamous cell carcinoma"):ti,ab,kw OR ("carcinoma"):ti,ab,kw OR ("adenosquamous carcinoma"):ti,ab,kw | 48140 |
| 27 | (CIN):ti,ab,kw | 1511 |
| 28 | (CIN1):ti,ab,kw | 136 |
| 29 | (CINI):ti,ab,kw | 284 |
| 30 | (CINII):ti,ab,kw | 13 |
| 31 | (CIN2):ti,ab,kw | 403 |
| 32 | (CIN3):ti,ab,kw | 230 |
| 33 | (CINIII):ti,ab,kw AND ("CIN III"):ti,ab,kw | 2 |
| 34 | ("cervical intraepithelial neoplasia"):ti,ab,kw | 956 |
| 35 | #14 or #15 or #16 or #17 or #18 or #19 or #20 or #21 or #22 or #23 or #24 or #25 or #26 or #27 or #28 or #29 or #30 or #31 or #32 or #33 or #34 [cervical pre-cancer or cancer] | 250803 |
| 36 | ("human papilloma virus"):ti,ab,kw OR ("human papilloma viruses"):ti,ab,kw | 381 |
| 37 | (hpv):ti,ab,kw OR ("papillomavirus"):ti,ab,kw OR ("papilloma virus infection"):ti,ab,kw OR ("papilloma virus"):ti,ab,kw | 4114 |
| 38 | #36 or #37 [HPV] | 4114 |
| 39 | #35 or #38 [cervical precancer/cancer + HPV] | 251917 |
| 40 | #13 and #39 [FGS + HPV or cervical precancer/cancer) | 2 |

*Cochrane Databases of Systematic Reviews; Central Trials – current content July 1998 (first published content 1908); Cochrane Clinical Answers November 2012; Cochrane Editorials March 2010

|  | **Global Index Medicus**   \| Database name \| Global Index Medicus \| \| --- \| --- \| \| Dates of database coverage \| 1957 to 2023 Week 10 \| \| Date searched \| 5 April 2023 \| \| Searched by \| AS \| \| Number of results \| 178 \| \| Search strategy notes \| Search lines ending in a ‘/’ are subject heading searches. Search lines beginning ‘exp’ are exploded subject heading searches. Search lines ending in .ti,ab. search in the title and abstract only. or/*x-y* combines search sets in the range *x-y* with Boolean operator OR. * is used for truncation of words. adj*n* searches for words within *n* words of each other. \|  \| 1 \| (tw:(Schistosoma haematobium)) \| 437 \| \| --- \| --- \| --- \| \| 2 \| (tw:(Schistosomiasis)) \| 5845 \| \| 3 \| (tw:(Schistosoma )) \| 5790 \| \| 4 \| (tw:(Bilharz*)) \| 4466 \| \| 5 \| 1 or 2 or 3 or 4  ((Bilharz*) or (Schistosoma ) or (Schistosomiasis) or (Schistosoma haematobium)) \| 7621 \| \| 6 \| (tw:(Female genital)) OR (tw:(Genitalia, female)) \| 7,184 \| \| 7 \| (tw:(Female genital system)) \| 1,490 \| \| 8 \| (tw:(Female genital tract)) \| 1,260 \| \| 9 \| (tw:(cervic*)) \| 43,204 \| \| 10 \| (tw:(cervix )) OR (tw:( Cervix Uteri)) \| 11,542 \| \| 11 \| (tw:(Cervico*)) \| 11,018 \| \| 12 \| 6 or 7 or 8 or 9 or 10 or 11   ((Female genital or Genitalia, female) or (Female genital system) or (female genital tract) or (cervic*) or (cervix or cervix uteri) or (cervico*)) \| 631, 712 \| \| 13 \| 5 and 12  ((Bilharz*) or (Schistosoma ) or (Schistosomiasis) or (Schistosoma haematobium)) AND ((Female genital or Genitalia, female) or (Female genital system) or (female genital tract) or (cervic*) or (cervix or cervix uteri) or (cervico*)) \| 1899 \| \| 14 \| (tw:(Genital Neoplasms, Female)) \| 1354 \| \| 15 \| (tw:(Cervical Intraepithelial Neoplasia)) OR (tw:(Uterine Cervical Neoplasms)) \| 8167 \| \| 16 \| (tw:(Uterine Cervical Dysplasia)) OR (tw:(Uterine Cervical Diseases)) OR (tw:(cervical)) \| 40,022 \| \| 17 \| (tw:(Precancerous Conditions)) OR (tw:( precancer*)) \| 2414 \| \| 18 \| (tw:(pre-cancer*)) \| 1019 \| \| 19 \| (tw:(premalignant)) \| 1079 \| \| 20 \| (tw:(neoplas*)) \| 174,640 \| \| 21 \| (tw:(dysplas*)) \| 12717 \| \| 22 \| (tw:(dyskaryosis)) \| 12 \| \| 23 \| (tw:(Squamous*)) \| 20,239 \| \| 24 \| (tw:(cancer)) OR (tw:(Neoplasm*)) \| 231,915 \| \| 25 \| (tw:(“Cancerous”)) \| 1776 \| \| 26 \| (tw:(Carcinoma, Squamous Cell)) OR (tw:(Carcinoma, Adenosquamous)) OR (tw:(Carcinoma)) \| 328 \| \| 27 \| (tw:(CIN)) \| 1742 \| \| 28 \| (tw:(CIN1)) \| 705 \| \| 29 \| (tw:(CINI)) \| 39 \| \| 30 \| (tw:(CIN2)) \| 781 \| \| 31 \| (tw:(CINII)) \| 41 \| \| 32 \| (tw:(CIN3)) \| 665 \| \| 33 \| (tw:(CINIII*)) \| 35 \| \| 34 \| (tw:(Cervical intraepithelial neoplasia)) \| 1710 \| \| 35 \| 14 or 15 or 16 or 17 or 18 or 19 or 20 or 21 or 22 or 23 or 24 or 25 or 26 or 27 or 28 or 29 or 30 or 31 or 32 or 33 or 34  ((Genital Neoplasms, Female) or (Cervical Intraepithelial Neoplasia) OR (Uterine Cervical Neoplasms) or (Uterine Cervical Dysplasia) OR (Uterine Cervical Diseases) OR (cervical) or (Precancerous Conditions) OR ( precancer*) or (pre-cancer*) or (premalignant) or (neoplas*) or (dysplas*) or (dyskaryosis) or (Squamous*) or (cancer) OR (Neoplasm*) or (“Cancerous”) or (Carcinoma, Squamous Cell) OR (Carcinoma, Adenosquamous) OR (Carcinoma) or (CIN) or (CIN1) or (CINI) or (CIN2) or (CINII) or (CIN3) or (CINIII*) or (Cervical intraepithelial neoplasia)) \| 293,966 \| \| 36 \| ((tw:(HPV)) OR (tw:(Papillomavirus Infections)) OR (tw:(Papillomaviridae)) \| 159,741 \| \| 37 \| tw:(human papillomavirus) \| 4538 \| \| 38 \| 36 or 37 (hpv)  ((HPV) OR (Papillomavirus Infections)) OR (Papillomaviridae) OR (human papillomavirus) \| 224966 \| \| 39 \| 35 or 38 (hpv or cervical precancer/cancer)   ((HPV) OR (Papillomavirus Infections) OR (Papillomaviridae) OR (human papillomavirus)) OR ((Genital Neoplasms, Female) or (Cervical Intraepithelial Neoplasia) OR (Uterine Cervical Neoplasms) or (Uterine Cervical Dysplasia) OR (Uterine Cervical Diseases) OR (cervical) or (Precancerous Conditions) OR ( precancer*) or (pre-cancer*) or (premalignant) or (neoplas*) or (dysplas*) or (dyskaryosis) or (Squamous*) or (cancer) OR (Neoplasm*) or (“Cancerous”) or (Carcinoma, Squamous Cell) OR (Carcinoma, Adenosquamous) OR (Carcinoma) or (CIN) or (CIN1) or (CINI) or (CIN2) or (CINII) or (CIN3) or (CINIII*) or (Cervical intraepithelial neoplasia)) \| 295343 \| \| 40 \| 13 and 39 (fgs and [hpv or cervical precancer/cancer]) \| 178 \| |
| --- | --- | --- | --- | --- | --- | --- | --- | --- | --- | --- | --- | --- | --- | --- | --- | --- | --- | --- | --- | --- | --- | --- | --- | --- | --- | --- | --- | --- | --- | --- | --- | --- | --- | --- | --- | --- | --- | --- | --- | --- | --- | --- | --- | --- | --- | --- | --- | --- | --- | --- | --- | --- | --- | --- | --- | --- | --- | --- | --- | --- | --- | --- | --- | --- | --- | --- | --- | --- | --- | --- | --- | --- | --- | --- | --- | --- | --- | --- | --- | --- | --- | --- | --- | --- | --- | --- | --- | --- | --- | --- | --- | --- | --- | --- | --- | --- | --- | --- | --- | --- | --- | --- | --- | --- | --- | --- | --- | --- | --- | --- | --- | --- | --- | --- | --- | --- | --- | --- | --- | --- | --- | --- | --- | --- | --- | --- | --- | --- | --- | --- | --- | --- | --- |

1. Falconer, Jane, Removing duplicates from an EndNote library. Library & Archives Service Blog: London School of Hygiene & Tropical Medicine. 2018. [online blog] <http://blogs.lshtm.ac.uk/library/2018/12/07/removing-duplicates-from-an-endnote-library/>. [↑](#footnote-ref-1)
