# Supplementary material for: Association of female genital schistosomiasis and human papillomavirus and cervical pre-cancer: a systematic review
Source: BMC Womens Health. 2025 Jan 3;25:2. doi: 10.1186/s12905-024-03514-0 (PMC11697648; doi:10.1186/s12905-024-03514-0)
Supplement: Supplementary file 2 — Supplementary Material 2. [file 12905_2024_3514_MOESM2_ESM.doc]

**Quality assessment using Newcastle – Ottawa assessment scale for cross-sectional and cohort studies for the manuscript entitled “Association of female genital schistosomiasis and human papillomavirus and cervical precancer: a systematic review”**

Description of the grading system

For cohort studies (evaluating HPV persistence or cervical cancer incidence), a study can be awarded a maximum of one star for each numbered item within the Selection (total of 4 items). There are three items in the Outcome category (worth a total of 4 stars) categories. A maximum of two stars can be given for Comparability, allowing for maximum stars=10.

| **Study** | **Selection** | | | | **Comparability of the Exposed and Non-exposed Cohort** | **Outcome** | | | **Total** |
| --- | --- | --- | --- | --- | --- | --- | --- | --- | --- |
|  | Representativeness of the exposed cohort  **Max=1 star** | Selection of the non-exposed cohort  **Max=1 star** | Ascertainment of exposure  **Max=1 star** | Outcome not present at baseline  **Max=1 star** | Adjustment for confounders  **Max=2 stars** | Assessment of outcome  **Max=2 stars** | Adequate Duration of Follow-Up  **Max=1 star** | Adequacy of Follow-Up  **Max=1 star** | Max =10 |

For cross-sectional studies (evaluating prevalence of high-risk human papillomavirus (HR-HPV), atypical cells of undetermined significance or cervical intraepithelial neoplasia (ASCUS-CIN1+), high grade squamous intraepithelial neoplasia (HSIL-CIN2+), or cervical cancer), a study can be awarded a maximum of one star for each numbered item within the Selection criteria (total of 4 items). There are two items in the Outcome category (worth a total of 3 stars). A maximum of two stars can be given for Comparability, allowing for maximum stars=9.

| **Study** | **Selection** | | | | **Comparability of the Exposed and Non-exposed Cohort** | **Outcome** | | **Total** |
| --- | --- | --- | --- | --- | --- | --- | --- | --- |
|  | Representativeness of the exposed cohort  **Max=1 star** | Ascertainment of exposure  **Max=1 star** | Selection of the non-exposed cohort  **Max=1 star** | Sample size calculation  **Max=1 star** | Adjustment for confounders  **Max=2 stars** | Assessment of outcome  **Max=2 stars** | Statistical Methods  **Max=1 star** | Max =9 |

1. **Selection**
2. Representativeness of the Exposed Cohort

| Exposed Group | Score “1” | Score “0” |
| --- | --- | --- |
| Women who are living in *Schistosoma* endemic areas. | Women of reproductive age who are living in *Schistosoma* endemic areas | Women not of reproductive age who are living in an area non-endemic for schistosomiasis.  OR  Recruitment strategy vague or not well explained, difficulty in ascertaining representativeness of the cohort |

1. Selection of the Non-Exposed Cohort

| Exposure | Score “1” | Score “0” |
| --- | --- | --- |
| Women who are living in *Schistosoma* endemic areas. | Drawn from same source as women with FGS | Not drawn from same source as women with FGS |

1. Ascertainment of Exposure

| Exposure | Score “1” | Score “0” |
| --- | --- | --- |
| Women of reproductive age, post sexual debut who are living in *Schistosoma* endemic areas. | FGS, diagnosed by molecular diagnostics (techniques to identify *Schistosoma* DNA include: PCR, recombinase polymerase assay, or loop-mediated isothermal amplification).  OR  Detection of eggs through histopathological verification (including cervical cytology).  AND  A majority of the diagnostic tests used to diagnose FGS, which are used to calculate associations with the outcome, are molecular or biopsy based. | FGS diagnosed by visual methods (naked eye using vaginal speculum conducted by trained medical professional, or other method to magnify the cervix including traditional or hand-held colposcopy, or use of a digital camera).  OR  Self-report, no information, or unclear information. |

1. Demonstration that outcome of interest (HPV persistence and squamous intraepithelial lesion (SIL) was not present at the start of the study **(for cohort studies only)**.
   1. HPV Persistence
      1. For the outcome of HR-HPV persistence, a score of “1” is given if the study reported genotype specific persistence of HR-HPV (ie same genotype positive at baseline and follow-up).
      2. Score of “0” if the study defined HPV-persistence by “any HPV” positive at baseline and “any HPV” positive at follow-up, without distinction of genotype.
   2. Cervical precancer or squamous intraepithelial lesion
      1. For the outcome of cervical precancer or SIL incidence, a score of “1” is given if the study reported confirmed women without SIL at baseline.
      2. Score of “0” if cervical precancer or SIL ascertainment is not reported or unclear at baseline.
2. **Comparability**
3. Comparability of the Exposed and Non-exposed Cohort: Study controls for factors strongly predictive of prevalence of HR-HPV, ASCUS-CIN1+ , HSIL-CIN2+ or cervical cancer incidence by adjusting for confounders:
   1. score of “2” if study adjusts for any of: age, HIV status
   2. score of “1” if adjusts for sociodemographic (for example: water use behaviours) or reproductive health factors (such as: cervical cancer screening history, number of sexual partners)
   3. score of “0” if estimate is unadjusted for potential confounders (i.e. raw data used to estimate crude effect estimate, or original authors present crude effect estimate in univariate risk factor analysis)

**C. Outcome**

1. Assessment of Outcome

| Outcome | Score “2” | Score “1” | Score “0” |
| --- | --- | --- | --- |
| HR-HPV detection | Validated/commercial PCR or genotyping method; high risk defined using the current International Agency for Research on Cancer (IARC) classification: ‘carcinogenic to humans’ (HPV16, 18, 31, 33, 35, 39, 45, 51, 52, 56, 58, 59). The definition of HR can include ‘probable carcinogenic’ (HPV68), and HPV66 (targeted by some next generation HPV tests) | Definition of high risk includes “possible” carcinogenic types (26, 53, 66, 67, 70, 73, 82) or low risk types, or definition includes fewer than the 12 carcinogenic types | No information given  OR  Diagnosis by histological demonstration of koilocytosis or immunohistochemistry |
| ASCUS-CIN1+ or HSIL-CIN2+ detection | Directed biopsy indicated by colposcopy and/or following positive screen test in addition to random biopsy of normal looking quadrants (considered highest quality)  OR  Biopsy indicated by colposcopy or cytology abnormality and biopsy rate ≥75% | Histological verification of tissue obtained through directed biopsy indicated by colposcopy following abnormal cytology or HR-HPV positive screen test;  OR  A composite diagnosis of cytology + histology following colposcopy guided biopsy (with or without knowledge of cytology);  OR  Histological verification of tissue obtained through directed biopsy indicated by colposcopy without knowledge of cytology or HR-HPV test when biopsy rate >=50% | Cytology or colposcopy only without histological verification (irrespective of whether there was independent verification of cytology or colposcopy)  OR  Histological verification of tissue obtained through directed Biopsy indicated by colposcopy without knowledge of cytology or HR-HPV test when proportion of participants with biopsy <50% as this represents a very limited number of verified endpoints  OR  Use of VIA or VILI |
| Cervical Cancer Incidence | Histology confirmed, or ICD10 clinic records verified by histology | ICD10 clinic records | No information |

Duration of follow-up Long Enough for Outcomes to Occur **(Cohort Studies only)**

- 1. Cervical precancer or SIL:
     1. Score “0” if interval of follow up is less than ≤ 12 months; Score “1” if interval of follow up is longer than 12 months
  2. HPV (including HR-HPV) Persistence:
     1. Score “0” if interval of follow up is < 6 months
     2. Score “1” if interval of follow up is ≥ 6 months

1. Adequacy of Follow-up **(Cohort Studies only)**
   1. Score “1” if loss to follow up is ≤20% and full description of participants lost to follow-up, either in flowchart, or in table of descriptive characteristics
   2. Score “0” if loss to follow up is >20% and/or no information given
2. Statistical Methods **(Cross-Sectional Studies only)**
   1. Score “1” if the statistical test used to analyze the data is clearly described and appropriate, and the measurement of the association is presented, including confidence intervals and the probability level (p value). *
   2. Score “0” if the statistical test is not appropriate, not described or incomplete.

**Table 2. Risk of bias assessment of cohort studies evaluating the association of FGS and human papillomavirus and cervical precancer using a modified Newcastle Ottawa Scoring system**

| Study | Selection | | | | Comparability | Outcome | | | Total  **Max 10 stars** | Quality  (1-3 low; 4-7 moderate; 8-10 high) |
| --- | --- | --- | --- | --- | --- | --- | --- | --- | --- | --- |
|  | Representativeness of the exposed  **Max 1 star** | Selection of the non-exposed  **Max 1 star** | Ascertainment of exposure  **Max 1 star** | Outcome not present at study onset  **Max 1 star** | Adjustment for confounders  **Max 2 stars** | Assessment of outcome  **Max 2 stars** | Duration of follow-up  **Max 1 star** | Adequacy of follow-up  **Max 1 star** |  |  |
| Kjetland 2010 – Any HPV Persistence | 1 star | 1 star | 0 stars | 0 stars | 0 stars | 0 stars | 0 stars | 0 stars | 2 stars | LOW |
| Kjetland 2010 – Cervical Precancer | 1 star | 1 star | 0 stars | 0 stars | 1 star | 0 star | 1 star | 1 star | 5 stars | MODERATE |

**Table 3. Risk of bias assessment of cross - sectional studies evaluating the association of FGS and human papillomavirus and cervical precancer using a modified Newcastle Ottawa Scoring system**

| Study | Selection | | | | Comparability | Outcome | | Total  **Max 9 stars** | Quality  (1-3 low; 4-7 moderate; 8-9 high) |
| --- | --- | --- | --- | --- | --- | --- | --- | --- | --- |
|  | Representativeness of the exposed  **Max 1 star** | Selection of the non-exposed  **Max 1 star** | Ascertainment of exposure  **Max 1 star** | Sample size calculation  **Max 1 star** | Adjustment for confounders  **Max 2 stars** | Assessment of outcome  **Max 2 stars** | Statistical Methods  **Max 1 star** |  |  |
| Kjetland 1996 – Cervical Precancer | 1 star | 1 star | 1 star | 0 stars | 0 stars | 1 star | 1 star | 5 stars | MODERATE |
| Kjetland 1996 – SCA | 1 star | 1 star | 1 star | 0 stars | 0 stars | 2 stars | 1 star | 6 stars | MODERATE |
| Kjetland 2010 – HR HPV Prevalence | 1 star | 1 star | 0 stars | 0 stars | 2 stars | 2 stars | 1 star | 7 stars | MODERATE |
| Pillay 2016 – Cervical Precancer | 0 stars | 1 star | 1 star | 0 stars | 0 stars | 0 stars | 0 stars | 2 stars | LOW |
| Rafferty 2021 - VIA | 1 star | 1 star | 1 star | 0 stars | 2 stars | 0 stars | 1 star | 6 stars | MODERATE |
| Kutz 2023 – Any HPV Prevalence | 1 star | 1 star | 0 stars | 0 stars | 1 star | 2 stars | 1 star | 6 stars | MODERATE |
| Shukla 2023 – Any HPV Prevalence | 0 stars | 1 star | 0 stars | 1 star | 2 stars | 2 stars | 1 star | 7 stars | MODERATE |

**Narrative summaries**

**Cohort Studies**

**Kjetland 2010**

Kjetland 2010 was set in rural Northwestern Zimbabwe. Women included in the prospective study were recruited as part of a 2005 cross-sectional study that enrolled 527 rural women between 15 and 49 years of age (median age not given) between October 1998 and March 1999 [1]. In the 2005 analysis, of 557 women tested, 236 (42%) were positive for any HPV type, and 54 (23%) tested positive for HR-HPV. The initial cross-section study included 236 consenting women between 15 and 49 years of age (median age not given) [1]. Women taking part in the baseline study lived within a 9- kilometer radius of a clinic built for the study [1]. Study recruitment followed the course of the river and the authors report “women were recruited from the area immediately around the clinic of Mupfure from three pick-up points and from the area around these pick-up points”, though no formal recruitment strategy is described. This study was set in an endemic area among women of reproductive age. Overall, the population is likely to be representative of women who acquire FGS and HPV who then may be at risk for persistent HPV infection and consequent pre-cancer (score “1”, category *selection*, subcategory: *representativeness of the exposed cohort*). The non-exposed cohort was selected from the same cohort as the exposed (score “1”, category *selection*, subcategory: *selection of the non-exposed cohort*). The exposure, FGS, was ascertained using colposcopy, PCR on cervicovaginal lavage (CVL), Papanicolau (pap) smear, and biopsy. From the baseline study [1], it appears that the majority of FGS diagnoses were made visually using a standard colposcope. However, in the 2010 follow-up study, the proportion of participants with colposcopy suggestive of FGS, cervical PCR, Pap smear and biopsy are not provided. Little information is given in the follow-up study [2] or the baseline study [1] regarding which diagnostic methods were used to obtain the total reported number of FGS cases (270/557, 49%) (score “0”, category *selection*, subcategory: *ascertainment of exposure*). Unfortunately, 321/557 (57.6%) of the study specimens from the baseline study [1] were destroyed in during Zimbabwean electricity cuts, allowing only 42.3% of the baseline participants to be eligible for follow-up study procedures (score “0” for category *outcome*, subcategory *adequacy of follow-up*). Additionally, cervicovaginal lavage was sample used for HPV detection, thus sensitivity to detect HPV may be reduced, and may be underestimated. The HPV testing method was well described, including the HPV types targeted. Cytology methods are not well-described. The categories of *selection*, subcategory: *outcome not present at study onset*; category *comparability*, subcategory: *adjustment for confounders*; category *outcome*, subcategory: *assessment of outcome*; category *outcome*, subcategory: *duration of follow-up* will be reviewed individual by outcome below. NOTE: the outcome ‘HR-HPV prevalence’ was cross-sectional and will be reviewed in the section on ‘Cross-sectional studies’.

**Outcome: HPV Persistence**

In the baseline study, HPV testing was performed in 42.3% (236/557) of the study population and any HPV was detected in 25.8% (61/236) and HR-HPV was detected in 54/236 (22%) of the baseline samples. Of the 54 HR-HPV samples that were not destroyed, 69% (37/54) with HR-HPV at baseline were sampled in the 5-year survey. The authors report “new HPV” as an outcome, but the overlap of any-HPV or low-risk HPV from the baseline cohort in the 54 participants with HR-HPV is not clearly described in the longitudinal cohort [2] (score “0” for category *selection*, subcategory: *outcome not present at study onset*). The HPV persistence outcome was reported numerically only with no statistical comparisons or effect estimates reported. Adjusting for confounders reduces the risk of bias in observational studies, since this study did not adjust for confounding variables on the association between FGS and HPV persistence, the possibility of bias cannot be excluded (score “0”, category *comparability*, subcategory: *adjustment for confounders*). The outcome of HPV persistence was assessed by HPV DNA detection via GP5+/6+ HPV PCR from CVL. After detection of HPV with a high-risk HPV probe cocktail, typing analysis for all HPV positive samples for high-risk types 16, 18, 26, 31, 33, 35, 39, 45, 51, 52, 53, 56, 58, 59, 66, 68, 73, and 82 was performed. However, the authors do not provide details about how individual low-risk HPV types were detected (score “0”, category *outcome*, subcategory: *assessment of outcome*). Some HPV infections would be expected to clear spontaneously by 18 months. HPV infections detected after five years may represent new and not persistent infections. Thus, the five-year duration of follow-up is inadequate to optimally assess HPV persistence (score “0”, category *outcome*, subcategory: *duration of follow-up*).

**Outcome: Cervical precancer (Squamous Intraepithelial Dysplasia)**

Biopsy in the baseline study [1] was performed only when the investigators were suspicious for malignancy. In the baseline study, Pap smears were performed in all participants and “investigated for cell atypia, and cervical intraepithelial neoplasia grades I−III (low to high grade squamous intraepithelial lesion [SIL]).” SIL was found in the baseline study in 6.8% of participants (36/527). However, in the 2010 study, the nomenclature changes and this outcome is described as “squamous intraepithelial dysplasia”. Since this outcome is not well-defined in the follow-up study [2], it is challenging to assess if the baseline and follow-up studies are referring to the same outcome. Additionally, ascertainment of the prevalence of SIL at baseline [1] is not clearly described among the 37 participants with HR-HPV who participated in follow-up (score “0”, category *selection*, subcategory: *outcome not present at study onset*) [2]. Adjusting for confounders reduces the risk of bias in observational studies. The association between HYSP (baseline) and squamous intraepithelial dysplasia was reported as a p-value without an effect size or adjustment for confounding. The authors describe that the model evaluating the association between HPV and HSIL was adjusted for age and HIV status. The effect size presented for the association between HSIL and FGS was adjusted for confounding, but the manuscript text is not explicit about what variables were adjusted for (aOR 7.1, 95% CI [0.5 – 92.1], p=0.1). The numbers available for this outcome were small but multivariable regression was performed for the association of FGS with squamous intraepithelial dysplasia. Additionally, while the effect size presented for the association between HYSP (follow-up) and squamous intraepithelial dysplasia is adjusted for confounding, the manuscript text is not explicit what variables were adjusted for (score “1”, category *comparability*, subcategory: *comparability of the exposed and non-exposed cohort*). To receive the highest score for outcome ascertainment with the use of cytology, the outcome of squamous intraepithelial dysplasia needs to assessed by histopathology after random cervical biopsies in the absence of pre-screening or HPV screening. Biopsies were only performed in 6% (32/527) of participants. Due to challenges with sample and smear preparation, Pap smears may be falsely negative. The imperfect sensitivity and specificity of cytology is a limitation when used exclusively to assess cervical pre-cancer (score “0”, category *outcome*, subcategory: *assessment of outcome*). The five-year duration of follow-up was adequate for the outcome of SIL to occur (score “1” for category *outcome*, subcategory: *duration of follow-up*).

**Cross Sectional Studies**

**Kjetland 1996**

Kjetland 1996 was set at Mangochi District Hospital in Malawi where the authors report that “*S. haematobium* is endemic in the area (p241)”, but do not provide a population prevalence [3]. Included in this cross-sectional study were 51 consenting women between 15 and 47 years of age (median 22 years). All participants were required to have urinary schistosomiasis (*S. haematobium* ova detected on a urine specimen) to qualify for study entry. Additionally, the women were recruited from a convenience sample of patients “of childbearing age who attended the hospital before lunch time, and who were willing to provide a urine sample” [3]. This study was set in an endemic area and recruited women of childbearing age and is felt to be representative of the population of women who could acquire FGS. Bias can be introduced in convenience sample recruitment, such as recruitment from a health facility, where the extremes of health or the under-resourced may not access health services (score “1”, category *selection*, subcategory: representativeness of the exposed cohort). The non-exposed cohort was selected from the same cohort as the exposed (score “1”, category *selection*, subcategory: *selection of the non-exposed cohort*). The exposure, FGS, was ascertained using colposcopy (proportions of positives not given), PCR on cervicovaginal lavage (CVL) (proportion positive for *Schistosoma* DNA not given), Papanicolau (Pap) smear (1/51, 1.9% positive for *S. haematobium* eggs), and biopsy (27/51, 54% positive for *S. haematobium* eggs). Histopathology is a highly specific means of FGS diagnosis since *S. haematobium* eggs are visualized in cervicovaginal tissue. In this publication, the majority of FGS cases in this publication were diagnosed by biopsy and then this proportion was used for determining associations with the outcome (score “1”, category *selection*, subcategory: *ascertainment of exposure*). No sample size calculations were performed, so certainty that the study was powered to evaluate the proposed outcomes is limited (score “0”, category *selection*, subcategory: *sample size calculation*). Adjusting for confounders reduces the risk of bias in observational studies, since this study did not adjust for confounding variables, the possibility of bias cannot be excluded (score “0”, category *comparability*, subcategory: *adjustment for confounders*). The outcome category (subcategories: assessment of outcome and adequacy of follow-up) will be assessed individually by outcome.

The outcome of SCA was assessed by histopathology after random cervical biopsies in the absence of pre-screening or HPV screening (score “2”, category *outcome*, subcategory: *assessment of outcome*). The SCA outcome was reported numerically only (one in the FGS group) with no statistical comparisons performed. However, given the small numbers, omitting effect size, confidence intervals, and p-value is appropriate (score “1” for category *outcome*, subcategory: *statistical methods*).

The cervical precancer outcome was assessed after fixing cervical smears which were examined with cytology. Since all participants underwent biopsy, there was histological verification of cytology outcomes (score “1”, category *outcome*, subcategory: *assessment of outcome*). The cervical precancer outcome was reported numerically only with no statistical comparisons performed. However, given the small numbers, omitting effect size, confidence intervals, and p-value is appropriate (score “1” for category *outcome*, subcategory: *statistical methods*).

**Pillay 2016**

Pillay 2016 was set in KwaZulu-Natal, South Africa where the authors report that the *Schistosoma* prevalence ranges between 40-98%. The prevalence of urinary schistosomiasis (*S. haematobium* eggs detected by urine microscopy) reported by the authors was 17.8% (71/394). This cross-sectional study enrolled 394 young women between 16 and 23 years of age (median age 19). Regarding sampling methods, the authors report, “as part of a school-based, clinical study of FGS, females from 42 randomly selected high schools were included”. Cervical precancer risk begins in persons without HIV around age 30 and the WHO recommends screening commence at this age. Thus, while this study was set in an endemic area and the participants were of reproductive age, the details of participant randomization within schools are vague and the age band included was not representative of the general population of women who can acquire FGS and cervical precancer (score “0”, category *selection*, subcategory: *representativeness of the exposed cohort*). The non-exposed cohort was selected from the same cohort as the exposed (score “1”, category *selection*, subcategory: *selection of the non-exposed cohort*). The exposure, FGS, was ascertained using colposcopy (proportions of positives not given), PCR on cervicovaginal lavage (CVL) (38/394, 9.6% positive for *Schistosoma* DNA), Papanicolau (Pap) smear (8/394, 2% positive for *S. haematobium* eggs). The majority of FGS cases used to determine associations with the outcome were diagnosed by molecular methods. PCR-based diagnostics are specific and a positive result reflects confirmation of a FGS diagnosis (score “1”, category *selection*, subcategory: *ascertainment of exposure*). No sample size calculations were performed (score “0”, category *selection*, subcategory, *sample size calculation*). In regards to the comparability of the exposed and non-exposed cohort, the authors report that squamous cell atypia (SCA) “was not significantly associated with any tests for schistosomiasis used in this study”. Adjusting for confounders reduces the risk of bias in observational studies, since this study did not adjust for confounding variables, the possibility of bias cannot be excluded (score “0”, category *comparability*, subcategory: *adjustment for confounders*). Due to challenges with sample and smear preparation, Pap smears may be falsely negative. The imperfect sensitivity and specificity of cytology is a limitation when used exclusively to assess cervical precancer and in this study the outcome of SCA was assessed by cytology only, without histological verification (score “0”, category *outcome*, subcategory: *assessment of outcome*). The statistical methods used to analyze the data for the reported SCA outcome were not clearly described and the SCA outcome was reported without effect size, confidence intervals, or p-value (score “0” for category *outcome*, subcategory: *statistical methods*).

**Kjetland 2010**

Please see the section above on ‘Cohort Studies’ where the Newcastle Ottawa scoring for the longitudinal outcomes for “HPV persistence” and “Cervical pre-cancer” from Kjetland 2010 are presented. The outcome ‘HR-HPV prevalence’, though captured in a cohort study, was cross-sectional and thus is scored in this section on cross-sectional studies. The ratings for the following categories are discussed in the section on ‘Cohort Study’ outcomes above: category *selection*, subcategory: *representativeness of the exposed cohort*; category *selection*, subcategory: *selection of the non-exposed cohort*; category *selection*, subcategory: *ascertainment of exposure;* category *outcome*, subcategory *adequacy of follow-up.* The categories of *selection*, subcategory: *sample size calculation*; category *comparability*, subcategory: *adjustment for confounders*; category *outcome*, subcategory: *assessment of outcome*; category *outcome*, subcategory: *statistical methods* will be reviewed below.

**Outcome: HR-HPV prevalence**

No sample size calculations were performed for the 2010 follow-up study (score “0”, category *selection*, subcategory, *sample size calculation*). Adjusting for confounders reduces the risk of bias in observational studies. The association of HR-HPV prevalence and FGS was adjusted for age. Adjusting for confounders minimizes the risk of bias in observational studies (score “2”, category *comparability*, subcategory: *adjustment for confounders*). The outcome of HR-HPV prevalence was assessed by HPV DNA detection via GP5+/6+ HPV PCR from CVL. After detection of HPV with a high-risk HPV probe cocktail, typing analysis for all HPV positive samples for high-risk types 16, 18, 26, 31, 33, 35, 39, 45, 51, 52, 53, 56, 58, 59, 66, 68, 73, and 82 was performed. Since cervicovaginal lavage was sample used for HPV detection, sensitivity to detect HPV may be reduced, and prevalence may be underestimated (score “2”, category *outcome*, subcategory: *assessment of outcome*). The statistical methods used in this study are appropriate (score “1” for category *outcome*, subcategory: *statistical methods*).

**Rafferty 2021**

Rafferty 2021 was set in Livingstone, Zambia. The prevalence of urinary schistosomiasis (*S. haematobium* eggs detected by urine microscopy) reported by the authors in this study was 6.3% (15/237). The prevalence of detectable circulating anodic antigen (CAA) was 14.8% (35/237). Rafferty 2021 recruited participants from the Population Cohort of HPTN 071 (PopART), a trial to measure the impact of an HIV combination prevention package, including universal HIV test and treat [4]. Trained community workers provided home visits to women who gave an “expression of interest” in the BILHIV study at the HPTN 071 (PopART) Population Cohort 36-month visit [4]. Due to high community HIV prevalence, all women regardless of age were routinely offered cervical cancer screening with visual inspection with acetic acid (VIA) at the cervical cancer clinic in Livingstone Central Hospital [5]. Rafferty 2021 was a sub-study of the BILHIV study [6] and was set in an *S. haematobium* endemic area and enrolled women ages 18-44 using probability sampling methods. However, it should be noted that not all women in the BILHIV study agreed to undergo voluntary cervical cancer screening. Cervical cancer data were available on 289/526 (54.9%) BILHIV study participants. This population is still be considered to be representative of the general population with FGS due to the use of probability-based sampling methods in women of reproductive age living in a *S. haematobium* endemic area, but readers should acknowledge there may be a risk of bias since only participants who agreed to additional screening are included (score “1”, category *selection*, subcategory: *representativeness of the exposed cohort*). The non-exposed cohort was selected from the same cohort as the exposed (score “1”, category *selection*, subcategory: *selection of the non-exposed cohort*). The exposure, FGS, was ascertained using hand-held colposcopy (70/237; 29.5% with characteristic FGS lesions), *Schistosoma* PCR on cervicovaginal lavage (CVL) or swabs (14/237, 5.9% *Schistosoma* DNA detected). FGS cases used to determine associations with the outcome were diagnosed by molecular methods. PCR-based diagnostics are specific and a positive result reflects confirmation of a FGS diagnosis (score “1”, category *selection*, subcategory: *ascertainment of exposure*). No sample size calculations were performed for the cervical precancer sub-study (score “0”, category *selection*, subcategory, *sample size calculation*). In regards to the comparability of the exposed and non-exposed cohort, the authors report that “multivariable logistic regression analysis was used to calculate adjusted odds ratios associations between VIA and FGS variables controlling for age category and HIV status as *a priori* confounders”. Adjusting for confounders minimizes the risk of bias in observational studies (score “2”, category *comparability*, subcategory: *adjustment for confounders*). The outcome of visual inspection with acetic acid (VIA) positivity was not assessed by any other method. The sensitivity of VIA is limited for the evaluation of cervical precancer (score “0”, category *outcome*, subcategory: *assessment of outcome*). The statistical methods in this study are well-described and appropriate (score “1” for category *outcome*, subcategory: *statistical methods*).

**Kutz 2023**

Kutz 2023 was set in three primary health care centers in Madagascar’s Marovay district [7]. In this manuscript the *S. haematobium* prevalence was not reported, but the authors do report that the Boeny region of Madagascar is known to be “highly endemic for *S. haematobium*”. This cross-sectional study enrolled 302 women between 18 and 49 years of age and the median age in the largest site, Marovoay, was 31 years (IQR 25.0 – 39.0). Regarding sampling methods, while being sexually active was not a criterion for inclusion, more than 90% of the participants had previously been pregnant. HPV acquisition begins after sexual debut, and all participants in this study were sexually active and are representative of the population that could acquire both HPV and FGS (score “1”, category *selection*, subcategory: *representativeness of the exposed cohort*). The non-exposed cohort was selected from the same cohort as the exposed (score “1”, category *selection*, subcategory: *selection of the non-exposed cohort*). The exposure, FGS, was ascertained using traditional colposcopy (62.6% (189/302) of the participants had colposcopy-defined FGS) and colposcopy-defined FGS was associated with the HPV outcome (score “0”, category *selection*, subcategory: *ascertainment of exposure*). A sample size calculation was not performed (score “0”, category *selection*, subcategory, *sample size calculation*). Adjusting for confounders reduces the risk of bias in observational studies, and this study adjusted for confounding variables, but what variables were adjusted for are not defined (score “1”, category *comparability*, subcategory: *adjustment for confounders*). The outcome of any HPV prevalence was assessed by HPV DNA detection via type specific E7 HPV PCR. The E7 assay utilizes HPV type specific primer pairs targeting the E7 region of 19 probable/possible high risk or high-risk HPV types (16, 18, 26, 31, 33, 35, 39, 45, 51, 52, 53, 56, 58, 59, 66, 68a and b, 70, 73, and 82 and 2 low risk HPV types (HPV 6 and 11). Since cervicovaginal lavage was sample used for HPV detection, sensitivity to detect HPV may be reduced, and prevalence may be underestimated (score “2”, category *outcome*, subcategory: *assessment of outcome*). The statistical methods used to analyze the data for the HPV outcome were clearly described and the association of visual-FGS was associated with the outcome (any HPV prevalence), which was reported with effect size, confidence intervals, and p-value (score “1” for category *outcome*, subcategory: *statistical methods*).

**Shukla 2023**

Shukla 2023 was set in KwaZulu-Natal, South Africa [8]. In this study the *S. haematobium* prevalence was 30.5% (256/840). This cross-sectional study enrolled 933 young women between 16 and 22 years of age (median age 18.7 years (standard deviation 1.6). Regarding sampling methods, the authors report, “as part of a school-based, clinical study of FGS, females from 42 randomly selected high schools were included”. HPV acquisition begins after sexual debut, and all participants in this study were sexually active, but the age band included was not representative of the general population of women who can acquire FGS and cervical precancer. Additionally, the details of participant randomization within schools are vague (score “0”, category *selection*, subcategory: *representativeness of the exposed cohort*). The non-exposed cohort was selected from the same cohort as the exposed (score “1”, category *selection*, subcategory: *selection of the non-exposed cohort*). The exposure, FGS, was ascertained using traditional colposcopy (22.5% (210/933) of the participants had colposcopy-defined FGS) and colposcopy-defined FGS was associated with the HPV outcome (score “0”, category *selection*, subcategory: *ascertainment of exposure*). A sample size calculation was performed (score “1”, category *selection*, subcategory, *sample size calculation*). Adjusting for confounders reduces the risk of bias in observational studies, and this study adjusted for age as a confounding variable (score “2”, category *comparability*, subcategory: *adjustment for confounders*). The outcome of any HPV prevalence was assessed by HPV DNA detection via GP5+/6+ HPV PCR. After detection of HPV with a high-risk HPV probe cocktail, typing analysis for all HPV positive samples for high-risk types 16, 18, 26, 31, 33, 35, 39, 45, 51, 52, 53, 56, 58, 59, 66, 68, 73, and 82 was performed. Since cervicovaginal lavage was sample used for HPV detection, sensitivity to detect HPV may be reduced, and prevalence may be underestimated (score “2”, category *outcome*, subcategory: *assessment of outcome*). The statistical methods used to analyze the data for the HPV outcome were clearly described and the association of colposcopy-FGS was associated with the outcome (any HPV prevalence), which was reported with effect size, confidence intervals, and p-value (score “1” for category *outcome*, subcategory: *statistical methods*).

**REFERENCES**
